# The Epstein-Barr virus deubiquitinase BPLF1 targets SQSTM1/p62 to inhibit selective autophagy

**DOI:** 10.1080/15548627.2021.1874660

**Published:** 2021-01-28

**Authors:** Päivi Ylä-Anttila, Soham Gupta, Maria G. Masucci

**Affiliations:** Department of Cell and Molecular Biology, Karolinska Institutet, Stockholm, Sweden

**Keywords:** Autophagy, deubiquitinase, EBV, large tegument protein, SQSTM1/p62

## Abstract

Macroautophagy/autophagy plays an important role in the control of viral infections and viruses have evolved multiple strategies to interfere with autophagy to avoid destruction and promote their own replication and spread. Here we report that the deubiquitinase encoded in the N-terminal domain of the Epstein-Barr virus (EBV) large tegument protein, BPLF1, regulates selective autophagy. Mass spectrometry analysis identified several vesicular traffic and autophagy related proteins as BPLF1 interactors and potential substrates, suggesting that the viral protein targets this cellular defense during productive infection. Direct binding of BPLF1 to the autophagy receptor SQSTM1/p62 (sequestosome 1) was confirmed by co-immunoprecipitation of transfected BPLF1 and by *in vitro* affinity isolation of bacterially expressed proteins. Expression of the catalytically active BPLF1 was associated with decreased SQSTM1/p62 ubiquitination and failure to recruit LC3 to SQSTM1/p62-positive aggregates. Selective autophagy was inhibited as illustrated by the accumulation of large protein aggregates in BPLF1-positive cells co-transfected with an aggregate-prone HTT (huntingtin)-Q109 construct, and by a slower autophagy-dependent clearance of protein aggregates upon transfection of BPLF1 in cells expressing a tetracycline-regulated HTT-Q103. The inhibition of aggregate clearance was restored by overexpression of a SQSTM1/p62[E409A,K420R] mutant that does not require ubiquitination of Lys420 for cargo loading. These findings highlight a previously unrecognized role of the viral deubiquitinase in the regulation of selective autophagy, which may promote infection and the production of infectious virus.

**Abbreviations:** BPLF1, BamH1 fragment left open reading frame-1; EBV, Epstein-Barr virus; GFP, green fluorescent protein; HTT, huntingtin; MAP1LC3/LC3, microtubule associated protein 1 light chain 3; PB1, Phox and Bem1 domain; PE, phosphatidylethanolamine; SQSTM1/p62, sequestosome 1; UBA, ubiquitin-associated domain

## Introduction

Macro-autophagy, commonly referred to as autophagy, is a conserved machinery for degradation and recycling of intracellular material that regulates the homeostasis of cells and tissues and promotes adaptation to changes in the microenvironment [1,2]. During autophagy, portions of the cytoplasm, protein aggregates, damaged/senescent organelles or invading pathogens are confined within double-membraned vesicles called autophagosomes and degraded following fusion of the autophagosomes with lysosomes. Dysfunction of autophagy is associated with diverse pathologies including neurodegenerative diseases, cancer, obesity, cardiovascular diseases, inflammatory and autoimmune disorders [3–5].

Several dozens of genes, collectively known as *ATG* (autophagy related) genes, are involved in autophagy [6]. The nucleation and assembly of the autophagosome membrane is a highly regulated process mediated by two protein complexes: the ULK1/2 complex comprising ATG13, RB1CC1/FIP200 and ATG101 that mediates signaling by MTOR (mechanistic target of rapamycin kinase) to the autophagic machinery, and the BECN1/Beclin 1-PIK3C3/VPS34 complex that consists of the class III phosphatidylinositol 3-kinase PIK3C3/VPS34 and its regulatory proteins PIK3R4/VPS15, BECN1 and ATG14 [7]. Together with other ATG proteins this complex promotes the elongation of phagophore membranes via activation of two sequentially acting ubiquitin-like conjugation systems that give rise to the ATG12-ATG5-ATG16L1 complex, which mediates the attachment of MAP1LC3/LC3 (microtubule associated protein 1 light chain 3) to phosphatidylethanolamine (PE) [8]. The lipidated form of LC3, known as LC3-II, is associated with the autophagosomal membranes and is commonly used as a marker of autophagy [9]. While nutrient deprivation-induced autophagy is non-selective, the selective autophagy of particular cargoes, such as damaged organelles and protein aggregates, is dependent on the engagement of specialized autophagy receptors, including OPTN (optineurin), CALCOC2/NDP52, SQSTM1/p62, BNIP3-BNIP3L/NIX factors or NBR1 [10]. These autophagy receptors bind to their targets through ubiquitin or LGALS (galectin) tags and to phagophore-anchored LC3 molecules via LC3-interacting regions (LIRs). Ubiquitin modification of both the core and regulatory components plays a key role in the positive and negative regulation of autophagic flux in both non-selective and selective autophagy [11].

Autophagy is a crucial cellular defense mechanism against invading pathogens and is particularly relevant during viral infections as viruses are obligate intracellular parasites whose successful replication requires the hijacking of many cellular functions. A growing body of evidence indicates that autophagy can exert both anti- and pro-viral activities depending on the virus, the type of infected cell and the cellular environment [12]. The antiviral effects often involve the selective recognition of incoming virus particles by autophagy receptors followed by engulfment and degradation (xenophagy) [13]. Core autophagy factors and regulators also participate in the control of viral replication and egress of viral particles and contribute to the initiation of both innate and adaptive immune responses [3,14]. Not surprisingly, many viruses have developed means to interfere with this cellular defense either by simply escaping autophagy [13] or by remodeling the autophagy process to promote their own replication and spread [14].

Epstein-Barr virus (EBV) is a gamma herpesvirus that infects human B lymphocytes and epithelial cells and is associated with a broad spectrum of malignancies including several types of non-Hodgkin B-cell lymphomas, T- and NK-cell lymphomas, and epithelial cancers of the nasopharynx and stomach [15]. Like other herpesviruses, EBV establishes both latent and productive infections in its host cells. Several EBV proteins expressed in latently infected cells were shown to be targets or regulators of autophagy. The EBV nuclear antigen (EBNA)-1 that resists proteasomal degradation [16] is processed by autophagy to inhibit apoptosis and maintain cell growth [17] and the Latent Membrane Protein (LMP)-1 induces autophagy to regulate its own turnover and reduce toxicity [18]. The function of BHRF1, the EBV encoded homolog of the autophagy inhibitor anti-apoptotic protein BCL2 [19], has not been explored but the homologs encoded by the Kaposi sarcoma virus (KSHV) and murine herpesvirus-68 (MH68) were shown to inhibit autophagy by forming stable complexes with BECN1 [20], which may impair BECN1-mediated tumor suppression and promote oncogenesis. Less is known about the regulation of autophagy during EBV replication. By monitoring autophagy markers and EBV lytic gene expression, De Leo et al. showed that autophagy is enhanced in the early phases of EBV lytic activation but decreases during the late phase [21]. Autophagic membranes were shown to be stabilized during EBV replication and LC3 was found in purified virus preparations, indicating that EBV subverts macro autophagy and uses autophagic membranes for secondary envelope acquisition during lytic infection [22]. Inhibition of autophagy was accompanied by a strong increase of lytic gene expression and enhanced virus yield suggesting the involvement of an early or late viral gene product but the viral product(s) responsible for the effects have not been identified.

Here we report that the deubiquitinase encoded in the N-terminal domain of the EBV large tegument protein BPLF1 interacts with several components of the vesicular trafficking and autophagy machinery, including the autophagy receptor protein SQSTM1/p62, and inhibits selective autophagy. This function may protect incoming virions during primary infection and inhibit the clearance of viral proteins during productive infection, thereby promoting the release of infectious viral particles.

## Results

### BPLF1 interacts with protein complexes involved in vesicular trafficking and autophagy

**Figure 1.**
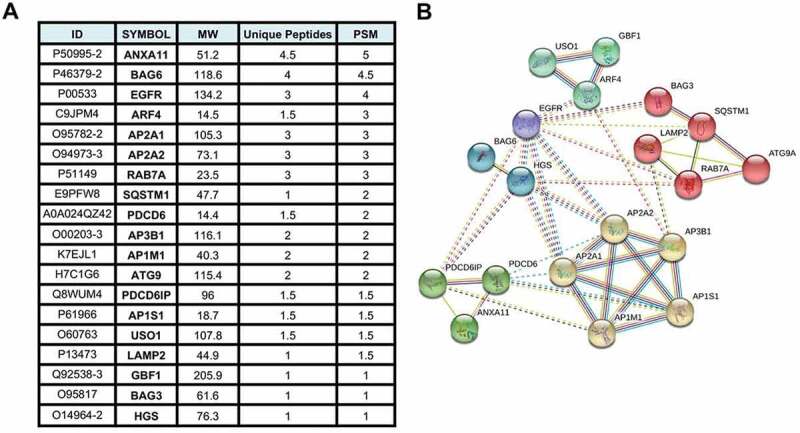
BPLF1-interacting proteins involved in autophagy related pathways identified by tandem mass spectrometry. (A) List of the 19 most abundant BPLF1 interacting proteins involved in autophagy identified by mass spectrometry. The values are derived from the mean of two independent experiments. ID = protein ID, MW = molecular weight, Unique Pep = number of unique peptides mapping to the protein, PSM = number of peptide spectrums matched to the protein. (B) Network diagram illustrating the interaction of the autophagy/vesicular transport related proteins enriched in the BPLF1 interactome. The network was created using STRING with Markov clustering algorithm (MCL) where each cluster is color annotated. The annotated interactions are color coded based on their source: curated databases (cyan line), experimentally determined (pink line), gene neighborhood (dark green line), gene fusions (red line), gene co-occurrence (dark blue), text mining (light green line), co-expression (black line) and protein homology (purple line)

Mass spectrometry analysis of proteins that co-precipitate with the N-terminal domain of EBV BPLF1 that is generated by CASP1 (caspase 1) cleavage in productively infected cells [23], identified 259 cellular proteins as potential interacting partners and putative substrates [24]. Based on Gene Ontology classification, the BPLF1 interactome is enriched in proteins involved in a variety of cellular functions, ranging from the cell cycle and RNA metabolism to innate immune response, suggesting that the viral enzyme has pleiotropic effects on the host cell remodeling that allows productive infection. Analysis of the protein interaction network identified a major interacting hub centering around several components of the vesicular trafficking and autophagy networks (Figure 1, Table S1). These included several subunits of the Adaptor Protein (AP)-1, AP-2 and AP-3 complexes that regulate vesicular transport from the plasma membrane and trans-Golgi networks [25], PDCD6 and PDCD6IP and ANXA11 (annexin A11) that are involved in ER-Golgi transport and cargo sorting [26], USO1 and the ARF family GTPases ARF4 and GBF1 that regulate vesicular trafficking [27], the only transmembrane component of the autophagy machinery ATG9 [6,28], the autophagy receptor SQSTM1/p62 [29], the BAG3 and BAG6 members of the BAG family of molecular chaperones [30] and LAMP2 (lysosomal associated membrane protein 2) glycoprotein that plays an important role in chaperone-mediated autophagy [31]. The large number of interacting partners suggests a possible role of the viral deubiquitinase in the regulation of vesicular trafficking and autophagy.

### BPLF1 binds to the autophagy receptor SQSTM1/p62 and regulates SQSTM1/p62 ubiquitination

**Figure 2.**
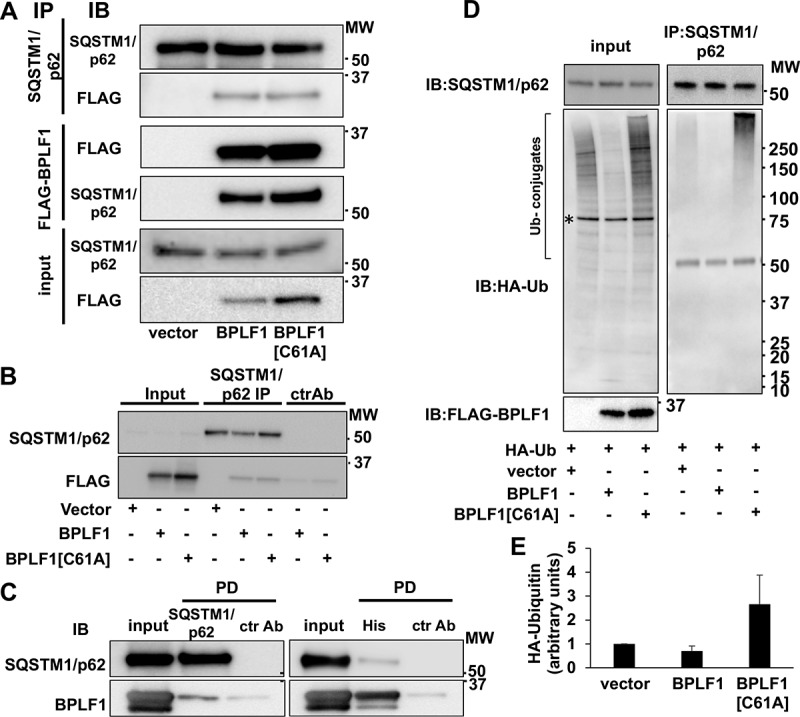
SQSTM1/p62 is a bona fide BPLF1 substrate. (A) Interaction of BPLF1 with the autophagy receptor protein SQSTM1/p62 was validated by reciprocal co-immunoprecipitation (IP). Western blots (WB) from representative experiments are shown. (B) Isotype control of SQSTM1/p62 immunoprecipitation. Weak non-specific binding of BPLF1 to the sepharose beads was observed with isotype control precipitation. (C) *In vitro* affinity isolation experiment shows direct binding of BPLF1 to SQSTM1/p62. (D) To resolve noncovalent protein interactions, endogenous SQSTM1/p62 was immunoprecipitated in denaturing conditions. Precipitated SQSTM1/p62 was free of ubiquitin in the presence of catalytically active BPLF1 and heavily ubiquitinated in the presence of the catalytic mutant. A non-specific 75 kDa band detected in total cell lysates by the anti-HA antibody is indicated by an asterisk. The antibody heavy chain is detected as a 50 kDa band in the IP blot. (E) Densitometry quantification of the ubiquitin smear in two independent experiments

Ubiquitination regulates the turnover and functions of several components of the autophagic machinery. In particular, the capacity of the selective autophagy receptor SQSTM1/p62 to target ubiquitinated cargo for autophagy was shown to be regulated by ubiquitination at Lys7, which inhibits the interaction of Lys7 with Asp69 and prevents oligomerization via the N-terminal Phox and Bem1 (PB1) domain [32,33], and Lys420, which promotes an open conformation of the C-terminal ubiquitin-associated (UBA) domain to allow cargo binding [34]. In order to investigate whether BPLF1 regulates SQSTM1/p62 ubiquitination, we first validated the interaction in HeLa cells transfected with FLAG-tagged versions of BPLF1 and the catalytic mutant BPLF1[C61A]. FLAG-tagged proteins were immunoprecipitated using anti-FLAG conjugated agarose beads while endogenous SQSTM1/p62 was immunoprecipitated using a specific antibody and Gamma-bind sepharose beads. The immunoprecipitates were immunoblotted and probed with SQSTM1/p62- and FLAG-specific antibodies (Figure 2A). Both wild type and catalytic mutant BPLF1 were detected in the SQSTM1/p62 immunoprecipitates (Figure 2A upper panels) and, conversely, SQSTM1/p62 was co-precipitated by FLAG-BPLF1 and FLAG-BPLF1[C61A] with similar efficiency (Figure 2A, middle panels). Although clearly weaker, *in vitro* affinity isolation assays performed with purified bacterially expressed recombinant BPLF1 and SQSTM1/p62 confirmed that the interaction is direct and independent of post-translational modifications that may occur in eukaryotic cells (Figure 2C).

To investigate whether SQSTM1/p62 is a substrate for the deubiquitinase activity of BPLF1, the levels of SQSTM1/p62 ubiquitination were compared in HeLa cells co-transfected with HA-tagged ubiquitin and either FLAG-BPLF1, FLAG-BPLF1[C61A] or, as control, the empty FLAG vector. Endogenous SQSTM1/p62 was immunoprecipitated under denaturing conditions to disrupt non-covalent interactions and immunoblots of the precipitates were then probed with specific antibodies (Figure 2D). Smears of high molecular weight species corresponding to poly-ubiquitinated SQSTM1/p62 were detected in the immunoprecipitates of vector-transfected cells. The ubiquitinated species were virtually absent in immunoprecipitates of cells expressing catalytically active BPLF1 while a strong accumulation of polyubiquitinated SQSTM1/p62 was reproducibly observed in cells expressing the catalytic mutant BPLF1[C61A] (Figure 2D right panel and Figure 2E).

**Figure 3.**
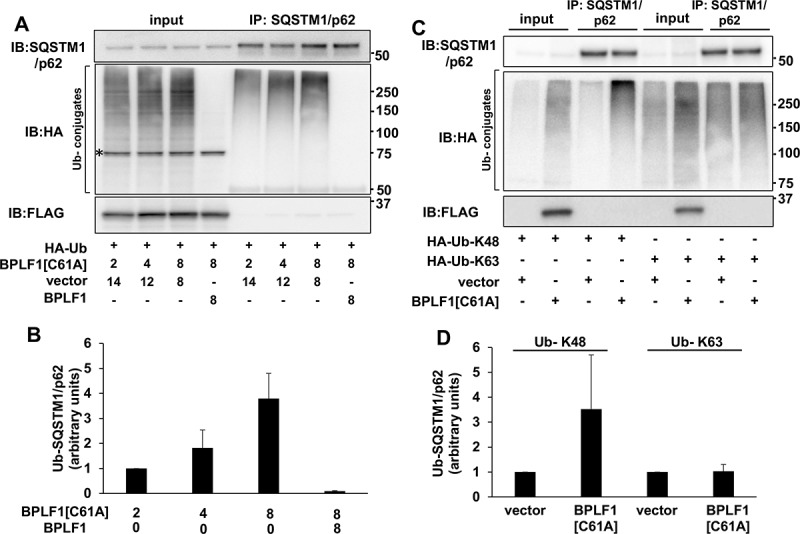
Catalytic mutant BPLF1 promotes SQSTM1/p62 hyper-ubiquitination. (A) SQSTM1/p62 was immunoprecipitated in denaturing conditions from cells expressing increasing amounts of BPLF1^C61A^ and HA-tagged ubiquitin and western blots were probed with an anti-HA antibody. The ubiquitination of SQSTM1/p62 was increased in the presence of BPLF^C61A^ in a dose dependent manner and reversed by spiking the transfections with catalytically active BPLF1. A non-specific 75 kD band detected in total cell lysates by the anti-HA antibody is indicated by an asterisk. (B) Quantification of the data presented in A. Mean ± SD of two experiments. (C) The hyper-ubiquitination of SQSTM1/p62 is largely due to addition of K48-linked ubiquitin chains. (D) Quantification of data presented in C; mean ± SD of two independent experiments

To further explore the capacity of catalytically inactive BPLF1 to promote SQSTM1/p62 ubiquitination, HeLa cells were co-transfected with HA-ubiquitin and increasing amounts of the BPLF1[C61A]-expressing plasmids. Endogenous SQSTM1/p62 was immunoprecipitated from cell lysates under denaturing conditions to disrupt non-covalent interactions and ubiquitinated SQSTM1/p62 species were detected by probing western blots with the anti-HA antibody (Figure 3A). Expression of the inactive BPLF1 was accompanied by a dose-dependent increase of SQSTM1/p62 ubiquitination that was reversed by spiking the transfection with equal amounts of the catalytically active enzyme (Figure 3A and B). Similar deubiquitination was observed by incubating immunoprecipitated SQSTM1/p62 with recombinant BPLF1 (Figure S1). BPLF1 trims both Lys48- and Lys63-linked polyubiquitin chains [24]. To investigate which type of chain conjugation is induced by the mutant BPLF1, HeLa cells were co-transfected with plasmids expressing FLAG-BPLF1[C61A] and HA-tagged ubiquitin mutants where all Lys residues except Lys63 (Ub-K63) or Lys48 (Ub-K48) were mutated to Arg. A reproducible increase in the levels of SQSTM1/p62 ubiquitination was observed in the presence of HA-Ub-K48, whereas there was no appreciable difference in the presence of HA-Ub-K63, suggesting that the catalytically inactive BPLF1 promotes the conjugation of Lys48-linked ubiquitin chains (Figure 3C, 3D). Collectively, these findings identify SQSTM1/p62 as an interacting partner and bona fide substrate of BPLF1, suggesting that the viral enzyme may play an important role in the regulation of selective autophagy during EBV infection.

### BPLF1 inhibits the recruitment of LC3 to SQSTM1/p62-positive structures

**Figure 4.**
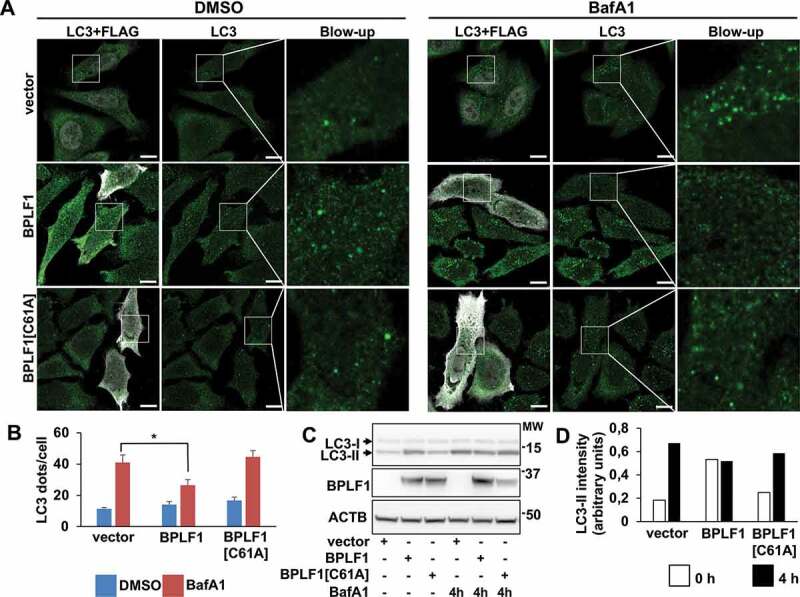
BPLF1 alters the autophagic flux. (A) Endogenous LC3 was detected by immunofluorescence in Hela cells transfected with either empty vector, BPLF1 or BPLF1[C61A]. Cells expressing catalytically active BPLF1 showed a small increase of LC3 puncta at steady state and significantly decreased accumulation of LC3 puncta upon treatment with BafA1. Scale bar: 10 μm. (B) Quantification of data presented in A; mean ± SEM of data pooled from three independent experiments. Statistical analysis was performed using Student t-test. *P ≤ 0.05. (C) Western blot illustrating the increase of lipidated LC3-II in cells expressing catalytically active BPLF1 and failure to accumulate LC3-II upon treatment with BafA1. (D) The intensity of the LC3-II bands was quantified by densitometry

**Figure 5.**
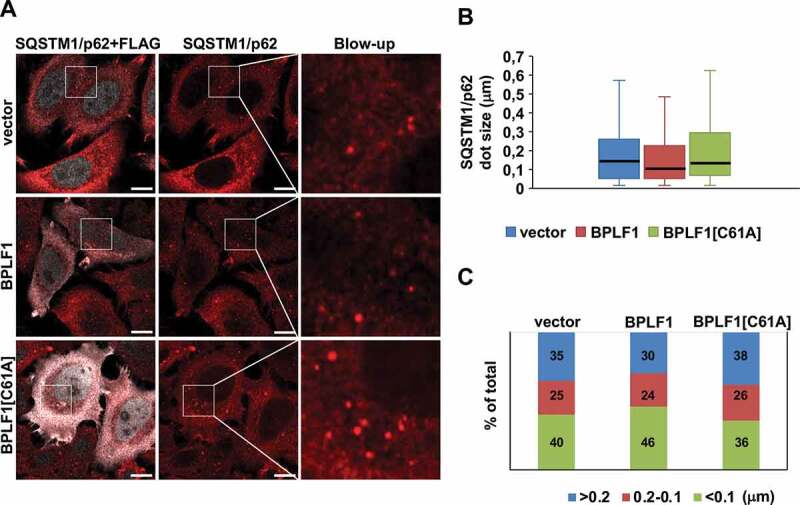
BPLF1 expression is associated with the formation of smaller SQSTM1/p62 aggregates. (A) Endogenous SQSTM1/p62 was detected by immunofluorescence in HeLa cells transfected with either empty vector, BPLF1 or BPLF1[C61A]. Scale bar: 10 μm. (B) Box and whiskers plot of the size of SQSTM1/p62 dots. A decrease in the median size of the SQSTM1/p62 dots was observed in the presence of catalytically active BPLF1. Median size: vector and BPLF1[C61A] = 0.140 μm; BPLF1 = 0.1 μm. (C) Small SQSTM1/p62 dots were overrepresented and large dots underrepresented in cells expressing the active viral enzyme. Figures D and E were constructed by pooling data from nine independent experiments

Polymerization of SQSTM1/p62 around polyubiquitinated cargo leads to the formation of protein bodies that, via interaction with LC3, are recruited to phagophores [35]. To investigate whether the binding to BPLF1 may affect the capacity of SQSTM1/p62 to serve as a cargo receptor for selective autophagy, we first asked whether the viral enzyme alters the localization of endogenous LC3 and SQSTM1/p62 (Figures 4 and 5). Although the magnitude of the effect varied considerably between experiments, a slight increase in the number of LC3-positive structures was observed under steady state conditions in cells expressing both catalytically active and inactive BPLF1 (Figure 4A, B). Expression of the active viral enzyme was associated with a significant reduction in the accumulation of LC3-positive dots following inhibition of lysosomal acidification by treatment with bafilomycin A_1_ (BafA1 Figure 4A, B), suggesting that the enzymatic activity may be involved in the regulation of autophagic flux. This was corroborated by analysis of the abundance of lipidated LC3-II in untreated and BafA1 treated cells. As illustrated by representative blot shown in Figure 4C and quantification of the specific bands in Figure 4D, a small increase of LC3-II was observed in untreated BPLF1-expressing and, to a minor extent, BPLF1[C61A]-expressing cells. As expected, treatment with BafA1 resulted in strong accumulation of LC3-II in vector-transfected cells, and comparable levels of accumulation were detected in cells expressing BPLF1[C61A]. In contrast, there was no further increase of LC3-II in cells expressing catalytically active BPLF1, supporting the conclusion that BPLF1 interferes with the autophagic flux.

**Figure 6.**
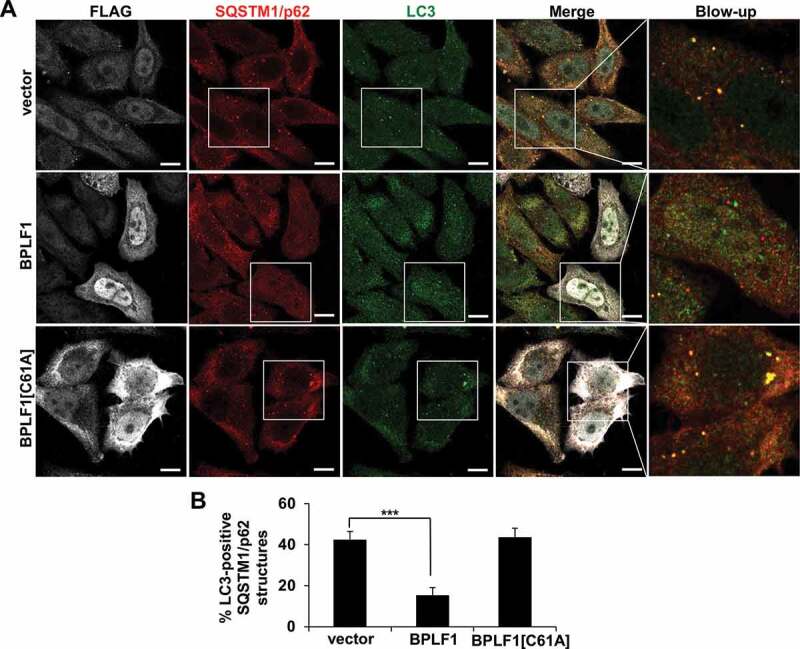
BPLF1 inhibits the recruitment of LC3 to SQSTM1/p62-positive structures. (A) HeLa cells expressing catalytically active or inactive BPLF1 or vector control were stained for endogenous SQSTM1/p62 and LC3. In cells expressing catalytically active BPLF1 a significant decrease of LC3 recruitment to SQSTM1/p62-positive structures was observed. (B) Quantification of the data presented in A. Mean ± SEM of the % LC3-positive SQSTM1/p62 dots detected in 14, 22 and 24 images pooled from two independent experiments. Statistical analysis was performed using Student t-test. ***P ≤ 0.001

In order to assess the relevance of this finding for selective autophagy we then turned to the effect of BPLF1 on SQSTM1/p62 localization. Staining of control and BPLF1-expressing cells with SQSTM1/p62-specific antibodies revealed no consistent differences in the number of SQSTM1/p62 aggregates, although also in this case the number of dots varied considerably between experiments (Figure 5A). However, as illustrated by the box and whiskers plot shown in Figure 5B and plotting of the distribution of dots in three size intervals shown in Figure 5C, small sized dots were consistently overrepresented in cells expressing catalytically active BPLF1. Furthermore, co-staining with SQSTM1/p62- and LC3-specific antibodies revealed a significantly decreased colocalization, suggesting that catalytically active BPLF1 prevents the recruitment of LC3 to SQSTM1/p62-positive aggregates (Figure 6A, B). A similar decrease of LC3 recruitment to SQSTM1/p62 structures was also observed in BPLF1-transfected U2OS cells (Figure S2) confirming that the effect is not cell-line specific. Thus, de-ubiquitination of SQSTM1/p62 by BPLF1 appears to affect both the type of aggregates and the targeting of SQSTM1/p62 aggregates to the autophagic machinery.

### BPLF1 regulates the clearance of HTT-polyQ aggregates

**Figure 7.**
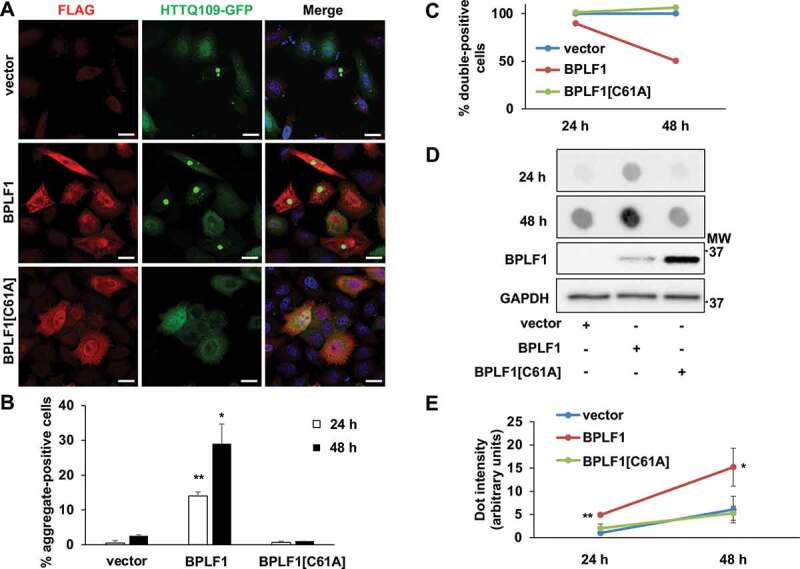
BPLF1 promotes the formation of HTTQ109 aggregates in transfected cells. HeLa cells were co-transfected with HTTQ109-GFP and BPLF1, BPLF1[C61A] or empty vector and expression of the transfected proteins was detected by immunofluorescence after 24 h or 48 h. (A) Representative micrographs illustrating the enhanced formation of HTTQ109-GFP aggregates in cells expressing catalytically active BPLF1. Scale bar: 10 μm (B) Quantification of number of aggregate-positive cells. Mean ± SEM of three independent experiments where a minimum of 176 cells were scored for each condition. Statistical analysis was performed using Student t-test. *P ≤ 0.05, **P ≤ 0.01. (C) The accumulation of toxic HTTQ109-GFP in cells expressing catalytically active BPLF1 induces cell death. The number of cells co-expressing HTTQ109-GFP and either BPLF1, BPLF1[C61A] or FLAG-vector was assessed 24 h or 48 h after transfection in three independent experiments. A selective loss of cells co-expressing HTTQ109-GFP and BPLF1 was observed after culture for 48 h. (D) Filter trap assays were performed with lysates of HeLa cells transfected as in A. A significantly stronger and progressive accumulation of HTTQ109-GFP aggregates was observed in cells expressing catalytically active BPLF1. The same lysates were fractionated by SDS-PAGE and immunoblotted to control for BPLF1 expression and loading. Images from one representative experiment out of three are shown. (E) Quantification of data presented in D; means ± SEM of three independent experiments

SQSTM1/p62 plays a key role in the selective autophagy that clears aggregate-prone proteins such as HTT mutants carrying extended poly-glutamine repeats [36]. In order to investigate whether the capacity of BPLF1 to modulate SQSTM1/p62 ubiquitination may lead to changes in selective autophagy, HeLa cells were co-transfected with plasmids expressing an aggregation-prone HTT mutant containing 109 glutamines fused to green fluorescent protein (HTTQ109-GFP) together with FLAG-BPLF1, FLAG-BPLF1[C61A] or control empty vector. Fluorescent aggerates were detected in 0.5% of the cells transfected with HTTQ109-GFP and the empty vector 24 h after transfection, and a small increase of positive cells (from 0.5 to 2.5%) was observed after 48 h (Figure 7A, B). The percentage of aggregate-positive cells was significantly increased upon co-expression of the catalytically active BPLF1 (14% after 24 h and 29% after 48 h) while expression of the inactive mutant BPLF1[C61A] had no appreciable effect (0.7% of cells after 24 h and 1% after 48 h). The effect of BPLF1 on the formation of HTTQ109 aggregates is likely to be underestimated since, in line with the expected toxicity of the aggregates [37], the recovery of HTTQ109/BPLF1 double-positive cells was strongly reduced compared to HTTQ109/vector- or HTTQ109/BPLF1[C61A]-transfected cells (Figure 7C). A filter trap assay was then used to assess the abundance of HTTQ109 aggregates. Total cell lysates were blotted on cellulose acetate membrane for aggregate trapping using a dot-blot apparatus and parallel lysates were fractionated by SDS-PAGE to control for total protein loading and BPLF1 expression. As illustrated by the representative blots shown in Figure 7D, and quantification of 3 independent experiments (Figure 7E), a time-dependent enhanced accumulation of HTTQ109 aggregates was detected in cells expressing wild type BPLF1 compared to vector transfected cells or cells expressing the catalytic mutant BPLF1[C61A], confirming that the active viral enzyme promotes the accumulation of HTTQ109 aggregates.

**Figure 8.**
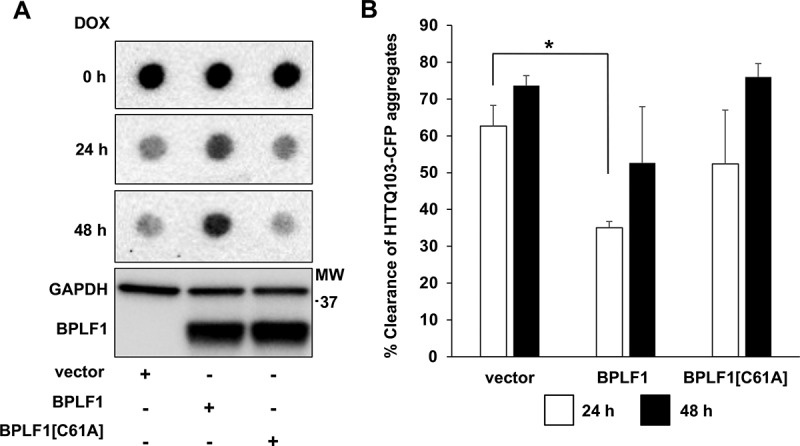
BPLF1 inhibits the clearance of HTTQ103 aggregates. HeLa HTTQ103-CFP cells were transfected with BPLF1, BPLF1[C61A] or empty vector and treated with doxycycline for indicated times to stop de novo synthesis of HTT and the clearance of HTTQ103-CFP aggregates was monitored by filter trap assay. The clearance of HTTQ103-CFP aggregates is inhibited in the presence of catalytically active BPLF1. The same lysates were fractionated by SDS-PAGE and immunoblotted to control for BPLF1 expression and loading. (A) Representative western blots illustrating the persistence of HTTQ103-CFP aggregates in cells expressing BPLF1. (B) The clearance of aggregates was calculated as the difference between the intensity of the dots at time 0 and the intensity after blocking de-novo synthesis by treatment with doxycycline for 24 h or 48 h. Expression of catalytically active BPLF1 resulted in significantly reduced aggregate clearance after 24 h while more variable effects were observed after 48 h due to toxicity of the HTTQ103-CFP aggregates. Means ± SEM of three independent experiments. Statistical analysis was performed using Student t-test. *P ≤ 0.05

In order to assess whether the formation of aggregates may be due to the capacity of BPLF1 to deubiquitinate HTTQ109, which could protect the soluble protein from proteasomal degradation, we took advantage of a previously described HeLa cell line that expresses an HTTQ103-CFP fusion protein under control of a Tet-off regulated promoter [36]. In these cells, withdrawal of tetracycline promotes the accumulation of HTTQ103-CFP aggregates that are promptly cleared by autophagy upon inhibition of de novo synthesis by tetracycline treatment, as confirmed by the failure to achieve clearance upon treatment with the autophagy inhibitor 3-methyladenine (3 MA) (Ref. 36 and Figure S3A, B). In line with the possibility that BPLF1 may act via inhibition of autophagy, filter trap assays performed to monitor the clearance of HTTQ103-CFP in cells expressing catalytically active or inactive BPLF1 revealed a significant delay in the clearance of HTTQ103-CFP aggregates upon expression of wild type BPLF1 while BPLF1[C61A] enzyme had no appreciable effect (Figure 8A, B). It is noteworthy that while the clearance of HTTQ103-CFP was significantly decreased in BPLF1-expressing cells after culture for 24 h, the effect was more variable after 48 h, probably due to the toxic effect of HTTQ103-CFP that would promote the selective death of cells that fail to clear the aggregates.

**Figure 9.**
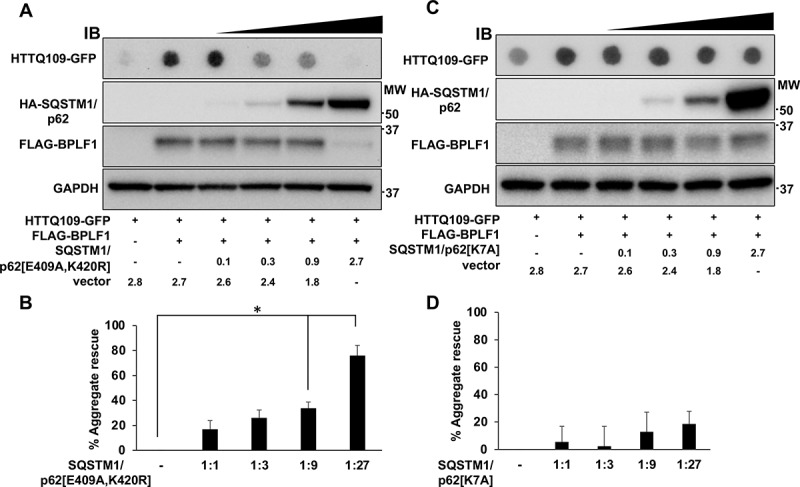
The SQSTM1/p62[E409A,K420R] mutant rescues the accumulation of HTTQ109-GFP aggregates induced by BPLF1. (A) HeLa cells were co-transfected with plasmids expressing HTTQ109-GFP, FLAG-BPLF1 and increasing amount of HA-SQSTM1/p62[E409A,K420R] and cell lysates collected after 48 h were analyzed in filter trap assays and immunoblotting. Representative immunoblots illustrating the capacity of HA-SQSTM1/p62[E409A,K420R] to rescue the accumulation of HTTQ109-GFP aggregates in a dose dependent manner. (B) Quantification of data presented in A; means ± SEM of three independent experiments. (C) Representative immunoblots illustrating the failure to rescue the accumulation of HTTQ109-GFP aggregates upon expression of SQSTM1/p62[K7A]. (D) Quantification of data presented in C; means ± SEM of three independent experiments. Statistical analysis was performed using Student t-test. *P ≤ 0.05

To investigate whether the inhibition of autophagy is a direct consequence of SQSTM1/p62 deubiquitination, we set out to rescue the clearance of aggregates by overexpressing a previously described SQSTM1/p62[E409A,K420R] mutant [34,38]. Ubiquitination of Lys420 regulates the capacity of the C-terminal UBA domain to interact with ubiquitinated cargo by preventing the formation of intermolecular UBA dimers [39]. Mutations that prevent the formation of hydrogen bonds between the Lys420 and Glu409 residues in the UBA domains of adjacent molecules renders SQSTM1/p62 constitutively open for cargo binding. HeLa cells were co-transfected with plasmids expressing the aggregation prone HTTQ109-GFP together with FLAG-BPLF1 and increasing amounts of HA-SQSTM1/p62[E409A,K420R]. Expression of the mutant rescued the BPLF1-induced accumulation of HTTQ109-GFP aggregates in a dose dependent manner (Figure 9A). Rescue was also observed upon overexpression of SQSTM1/p62 (Figure S3C, D), although higher amounts of the wild type protein were required for comparable levels of rescue (compare the Figure 9B and S3D). Overexpression of an autophagy defective SQSTM1/p62[K7A] mutant that cannot oligomerize via N-terminal PB1 domain failed to promote aggregate clearance (Figure 9B, C), suggesting that both exposure of the Lys7 residue via deubiquitination and opening of the UBA domain via K420 ubiquitination are required for efficient clearance of the aggregates. Interestingly, overexpression of SQSTM1/p62 and SQSTM1/p62[E409A,K420R] but not SQSTM1/p62[K7A] induced the degradation of BPLF1, (Figure 8A and S3C) suggesting that the viral proteins may be associated with aggregates that are targeted for autophagy. Taken together, these findings support the conclusion that BPLF1 regulates the clearance of autophagy cargo by deubiquitinating the cargo receptor SQSTM1/p62.

## Discussion

While the impact of autophagy on viral infections varies depending on the virus, the host cell and the phase of the virus cycle [12,40–42], compelling evidence points to a key role of selective autophagy in the clearance of both the incoming pathogen and newly synthesized viral proteins and underscores the capacity of viruses to effectively counteract this cellular defense to promote their own replication and spread. In this study, we report on the characterization of a new viral strategy for regulation of selective autophagy that relies on functional inactivation of the autophagy receptor SQSTM1/p62. We found that SQSTM1/p62 interacts with and is deubiquitinated by the ubiquitin deconjugase encoded in the N-terminal domain of the EBV large tegument protein BPLF1. This inhibits the colocalization of SQSTM1/p62 with LC3 decorated autophagic membranes and results in failure to clear protein aggregates.

BPLF1 is the EBV encoded member of a family of viral ubiquitin- and NEDD8-specific deconjugases that is conserved in all human and animal herpesviruses. The enzymes play multiple roles in infection by regulating the activity and turnover of proteins that control key cellular functions required for virus replication and infectivity as well as innate antiviral defenses [43]. By using an unbiased approach based on co-immunoprecipitation with the N-terminal catalytic domain of BPLF1, we have identified several components of the vesicular trafficking and autophagic machinery as potential interacting partners of BPLF1 (Figure 1). The interaction with proteins involved in ER and Golgi vesicular transport is in line with the known function of the large tegument proteins in virion assembly and secondary envelopment, leading to the production of infectious virus particles [44,45]. The contribution of the deconjugase activity to this step of the virus cycle is supported by the strongly reduced production of infectious virus in cells infected with recombinant viruses carrying inactivating mutations [46,47]. While the role of autophagy in herpesvirus replication and assembly and the viral products that interfere with the process are not fully understood, the findings that autophagy is suppressed during the late phase of EBV replication [48], that inhibition of autophagy by BECN1 knockdown or treatment with bafilomycin A_1_ enhances the expression of lytic antigens and the release of virus particles [21], and that LC3-containing autophagic membranes assist virus production and are incorporated in the EBV envelope [22] suggest that viral products expressed during the early/late phase of the productive cycle promote a broad reorganization of autophagy. This appears to involve both inhibition of the proteolytic function and boosting of the membrane remodeling capacity of the autophagic machinery. Our finding points to the enzymatic activity of BPLF1 as major contributor to this virus-induced remodeling.

We found that BPLF1 interacts with and deubiquitinates SQSTM1/p62 that plays a key role in selective autophagy by targeting ubiquitinated cargo to autophagic membranes. The interaction of BPLF1 with SQSTM1/p62 appears to be direct and independent of the catalytic function of BPLF1 (Figure 2A, B), although the relatively weaker affinity isolation observed with purified proteins suggests that additional binding partners may strengthen the interaction in cells. Interestingly, while ubiquitinated SQSTM1/p62 species were virtually undetectable in cells expressing the active viral enzyme, a strong and dose-dependent increase of ubiquitination was observed in cells expressing the catalytic mutant BPLF1[C61A] (Figure 2C, D and 3). Conceivably, one of several ligases that were shown to interact with BPLF1, including members of the cullin-ring (CRL) and tripartite motif (TRIM) ligase families [24,49], may be recruited to the complex resulting in the attachment of K48-linked ubiquitin chains on SQSTM1/p62. Although unlikely, we cannot formally exclude that binding of BPLF1 might displace a deconjugase that regulates the physiological levels of SQSTM1/p62 ubiquitination, as suggested by the recent finding that USP8 inactivates SQSTM1/p62 by promoting the detachment of Lys11-linked poly-ubiquitin chains from key residues in the UBA domain [50]. While outside of the scope of this investigation, a precise mapping of the sites of BPLF1-SQSTM1/p62 interaction and the identification of additional binding partners and Lys residues involved in the deubiquitination/ubiquitination events associated with expression of the viral enzyme will be required for a molecular understanding of the regulatory events.

Several E3 ligases have been implicated in the attachment of different types of ubiquitin chains to various Lys residues in SQSTM1/p62, both constitutively and in response to different cellular stressors [33,34,51,52]. Moreover, ubiquitin conjugating enzymes (E2) were shown to be sufficient for ubiquitination of SQSTM1/p62 under ubiquitin stress conditions [38], which further emphasizes the importance of this post-translational modification in the activity of the autophagy receptor. Amidst this complexity, ubiquitination sites located in the C-terminal UBA domain and N-terminal PB1 domain have emerged as important regulators of two key functions of the receptor, namely the capacity to bind ubiquitinated cargo, via the Lys420-ubiquitination-dependent display of binding sites in the UBA domain [34,38,39]; and the formation of oligomeric filaments that facilitates cargo sequestration by LC3 coated membranes, via deubiquitination of Lys7 in the PB1 domain [33,35,53]. Our findings offer important clues of how BPLF1 could interfere with these events. Analysis of LC3 localization in cells expressing catalytically active and inactive BPLF1 revealed a small steady state increase in the number of LC3-positive dots and accumulation of lipidated LC3-II in cells expressing the active enzyme, while, consistent with a mild inhibitory effect on the basal autophagic flux, the BafA1-induced accumulation of LC3 dots and LC3-II were impaired in cells expressing active BPLF1 (Figure 4A, B, C, D). The formation of SQSTM1/p62 aggregates was only marginally affected, although the size of the dots appeared consistently smaller in cells expressing the active enzyme (Figure 5A, B, C). In contrast, the colocalization of SQSTM1/p62 aggregates with LC3 was severely impaired in cells expressing catalytically active BPLF1 but unaffected in cells expressing the catalytic mutant (Figure 6, Figure S2). This, together with the formation of smaller aggregates, points to the C terminus of SQSTM1/p62 as a key target of the viral deubiquitinase. Indeed, deubiquitination of the C terminus would prevent the recruitment of cargo and the formation of large aggregates while smaller aggregates may still form via oligomerization of deubiquitinated PB1 domains. Accumulating evidence suggest that the cargo plays a key role in the nucleation of isolation membranes in selective autophagy [54]. Hence, the size of the aggregates may be critical for stable interaction with LC3 since SQSTM1/p62 contains only one LC3-interacting region (LIR), and oligomerization greatly amplifies the affinity of binding [35]. The possibility that the C-terminal domain of SQSTM1/p62 may be the primary target of BPLF1 is also supported by the failure of the catalytic mutant BPLF1[C61A] to prevent the colocalization of SQSTM1/p62 aggregates with LC3. It remains to be seen whether the enhanced ubiquitination induced by the mutant may compensate for a possible failure to promote oligomerization via the ubiquitinated N terminus.

While the effect of BPLF1 on SQSTM1/p62 ubiquitination and LC3 interaction points to inhibition of selective autophagy, the comparison of cells infected with EBV recombinant viruses expressing wild type and catalytic mutant BPLF1 would be required to assess whether this regulation occurs in infected cells under physiological levels of expression. We have previously reported on the production of HEK-293 cell lines carrying EBV recombinant viruses expressing wild-type and catalytic mutant BPLF1 that would be an ideal model for this analysis [24]. Unfortunately, the broad involvement of BPLF1 in multiple aspects of the productive virus cycle, and the failure of cells infected with the BPLF1-mutant virus to sustain full virus replication with variable expression of immediate early, early and late genes, precluded a meaningful comparison of the autophagic flux by biochemical assays. Fluorescence based analysis were also unreliable in the HEK-293-EBV cell background due to the peculiar growth pattern and massive cell detachment following induction of the productive cycle. To circumvent these technical limitations, we have investigated the effect of BPLF1 in a well-established model of selective autophagy. Mutant HTT containing expanded poly-glutamine repeats forms large aggregates of misfolded proteins that are degraded through SQSTM1/p62 mediated selective autophagy [36,55]. We found that expression of catalytically active BPLF1 promotes the formation of aggregates in cells transfected with a HTTQ109-expressing plasmid (Figure 7) and inhibits the clearance of preformed aggregates in cells expressing a tetracycline regulated HTTQ103 mutant (Figure 8). The interpretation of these findings is not straightforward since deubiquitination of soluble HTTQ109 may promote the formation of aggregates by preventing proteasomal degradation and deubiquitination of the aggregates could hamper their recognition by the autophagy receptor. Attempts to probe the ubiquitination of the aggregates by co-staining with ubiquitin antibodies were inconclusive due to the strong fluorescence of the GFP-tagged HTT mutant. Nevertheless, our study clearly supports the direct involvement of SQSTM1/p62 deubiquitination in the failure to clear preformed aggregates. We found that the inhibitory effect of BPLF1 could be rescued by overexpression of the SQSTM1/p62[E409A,K420R] mutant where loss of the inactivating interaction between Lys420 and Glu409 mimics the effect of ubiquitination (Figure 9). Thus, although deubiquitination of the cargo could contribute to the inhibitory effect of BPLF1, the level of ubiquitination appears to be sufficient for proficient interaction with the active SQSTM1/p62 receptor. Interestingly, rescue was also achieved by overexpression of wild type SQSTM1/p62, although less efficiently, whereas overexpression of the SQSTM1/p62[K7A] mutant had no effect. The lack of rescue by the SQSTM1/p62[K7A] mutant was expected since the Lys7 to Ala substitution inhibits intermolecular oligomerization of the PB1 domain, mimicking thereby the effect of ubiquitination. Yet, this finding confirms that rescue by the wild-type and E409A,K420R-mutant SQSTM1/p62 is not an artifact of overexpression, and further substantiate the key role of both Lys420 ubiquitination and Lys7 deubiquitination in regulating the receptor function of SQSTM1/p62. It is noteworthy that BPLF1 protein levels were strongly reduced upon overexpression of wild type and E409A,K420R-mutant SQSTM1/p62 while overexpression of SQSTM1/p62[K7A] had no effect (Figure 9A and Figure S3C). This suggests that BPLF1 is indeed recruited to a SQSTM1/p62 containing complex and, when the inhibitory activity is overwhelmed by either overexpression of the receptor or by expression of a constitutively active receptor, it may become itself a target of selective autophagy.

In conclusion, our findings identify the deubiquitinase activity of BPLF1 as a previously unrecognized viral regulator of selective autophagy. The large tegument proteins are expressed late in the productive virus cycle of herpesviruses when their capacity to inhibit selective autophagy could provide a means to restrict the degradation of abundantly produced proteins that are required for the assembly of infectious virus particles. In addition, the viral tegument is released to the host cytoplasm during primary infection. Inhibition of autophagy could allow the virus to escape immediate destruction and could also participate in the evasion of host innate immune responses that promotes the establishment of infection. Future work aiming to a precise characterization of the molecular interactions involved in the inhibition of autophagy may inspire novel strategies for interfering with infection.

## Materials and methods

### Chemicals

DL-dithiothreitol (DTT, D0632), N-ethylmaleimide (NEM, E1271), iodoacetamide (I1149), IGEPAL CA-630 (NP40, I3021), Triton X-100 (T9284), bovine serum albumin (BSA, A7906), sodium dodecyl sulfate (SDS, L3771), Tween-20 (P9416), ethylenediaminetetraacetic acid disodium salt dehydrate (EDTA, E4884), Trizma base (Tris, 93,349), 3-methyladenine (3 MA, M9281), bafilomycin A_1_ (B1793) and ciprofloxacin (I7850) were purchased from Sigma-Aldrich. Complete protease inhibitor cocktail (04693116001) and phosphatase inhibitor cocktail (04906837001) were purchased from Roche Diagnostic.

### Antibodies

Antibodies and their manufacturers were: rat anti-BPLF1 [56] (WB 1:1600, 2E5 supernatant) was from Helmholtz Zentrum (2E5); mouse anti-FLAG clone M2 (WB 1:4000, IF 1:400; F1804) and rabbit anti-LC3B (WB 1:1000, IF 1:200; L7543) were from Sigma-Aldrich; goat anti-FLAG (1:400; ab1257) and rabbit anti-SQSTM1/p62 (IP 1–2 μg/mg of lysate; ab101266), rabbit anti-GFP (WB 1:2000, ab290) and rabbit IgG isotype control (ab172730) were from Abcam; mouse anti-SQSTM1/p62 (WB 1:1000 IF 1:200; 610,832) was from BD Biosciences; rabbit anti-SQSTM1/p62 (WB 1:1000, 8025S) was from Cell Signaling Technology; mouse anti-HA clone 12 CA5 (WB 1:2000; 11,583,816,001) from Roche; mouse IgG isotype control (14–4732-82) was from Invitrogen; Alexa Fluor 488-, 555-, 594- and 647-conjugated secondary antibodies were from Thermo Fisher (A21206, A31570, A11032 and A21447, respectively).

### Plasmids

Plasmids encoding 3xFLAG-BPLF1 (amino acid residues 1–235), the catalytic mutant BPLF1[C61A], and HA-tagged ubiquitin were described previously [49].Plasmids pRK5-HA-UbK48 and pRK5-HA-UbK63 that encode for ubiquitin polypeptides where all Lys residues are mutated to Arg except for Lys48 or Lys63 were kindly provided by Harald Wodrich, Laboratoire de Microbiologie Fondamentale et Pathogenicite UMR-CNRS, University of Bordeaux. HTTQ109-GFP plasmid was a gift from Nico Dantuma, Department of Cell and Molecular Biology, Karolinska Institutet. The coding sequence of the SQSTM1/p62[K420R/E409A] mutant was excised from the plasmid pET28a-p62[_K420R/E409A]_-Flag [38] kindly provided by Ronggui Hu (Institute of Biochemistry and Cell Biology, Shanghai Institutes for Biological Sciences, China) and inserted between HindIII and NotI restriction sites of the HA-SQSTM1/p62 expression vector [57] (Addgene, 28,027; deposited by Qing Zhong). The HA-SQSTM1/p62[K7A] mutant was made from the HA-SQSTM1/p62 construct using the QuickChange II XL site-directed mutagenesis kit (Agilent Technologies, 200,521) using the primers: p62-K7A forward 5ʹ-cgtcgctcaccgtggcggcctaccttctgg-3ʹ reverse 5ʹ-ccagaaggtaggccgccacggtgagcgacg-3ʹ.

### Cell lines and transfection

HeLa cells (ATCC RR-B51S), and U2OS (ATCC HTB-96) cells were cultured in Dulbecco’s modified Eagle’s medium (DMEM, Sigma-Aldrich, D6429), supplemented with 10% FCS (Gibco-Invitrogen, 10,270–106), and 10 μg/ml ciprofloxacin, and propagated at 37°C in a 5% CO_2_ incubator. HeLa cells expressing mutant HTT (HeLa exon1 HTT-103QmCFP) [36] (gift from Ai Yamamoto, Department of Neurology, Columbia University) were cultured in the DMEM complete medium supplemented with 50 μg/ml geneticin and 100 μg/ml hygromycin for selection. HTT expression was inhibited with addition of 1–2 μg/ml doxycycline (Sigma-Aldrich, 44,577). Plasmid transfection was performed using the JetPEI (Polyplus transfection, 101–40 N) or Lipofectamine 2000 kits (Life technologies, 11,668,019) as recommended by the manufacturer.

### Tandem mass spectrometry and bioinformatics analysis

The mass spectrometry characterization of the BPLF1 interactome was previously reported [24]. For the current analysis, proteins that were absent in duplicate samples of immunoprecipitates from cells transfected with the FLAG-empty vector but were detected by at least one unique spectral count in both the FLAG-BPLF1 and FLAG-BPLF1[C61A] immunoprecipitates were considered as positive hits. The bioinformatics resource DAVID [58] was used to identify the overrepresentation of genes in particular functional categories. Pathways databases including Gene Ontology [59,60], Panther [61] and the Kyoto Encyclopedia of Genes and Genomes (KEGG) [62] were used for functional annotation. Functional interaction network analysis was performed using the Search Tool for the Retrieval of Interacting Genes (STRING) database v. 9.0 [63]. STRING integrates information on physical interactions and functional relationships identified by high-throughput biochemical analysis, mining of databases and literature, and prediction from genomic context analysis, into Protein-Protein-Interaction (PPI) networks.

### Immunoblotting and immunoprecipitation

For immunoblotting and co-immunoprecipitation cells harvested 24 h or 48 h post transfection were lysed in NP40 lysis buffer (50 mM Tris-HCl pH 7.6, 150 mM NaCl, 5 mM MgCl_2_, 1 mM EDTA, 1 mM DTT, 1% Igepal, 10% glycerol) supplemented with protease and phosphatase inhibitors and deubiquitinase inhibitors (20 mM NEM and 20 mM Iodoacetamide). Protein concentration was measured with a Lowry protein assay kit (Bio-Rad Laboratories). For co-immunoprecipitation, the cell lysates were incubated for 4 h with anti-FLAG agarose affinity gel (Sigma, A-2220). Precipitated complexes were washed with lysis buffer and eluted with FLAG peptide (Sigma, F4799) at a concentration of 400 μg/ml. For co-immunoprecipitation of endogenous SQSTM1/p62 the cell lysates were incubated for 2–4 h with specific antibody followed by 1–2 h with protein-G coupled Sepharose beads (GE Healthcare, 17–0885-01). To resolve protein complexes for denaturing immunoprecipitation, cell pellets were lysed in 100 μl NP-40 lysis buffer supplemented with 1% sodium dodecyl sulfate (SDS). Before immunoprecipitation NP-40 buffer was added to reach a final concentration of 0.1% SDS. Immunocomplexes were washed with lysis buffer and elution was performed by boiling for 5 min in 2x SDS-PAGE loading buffer. Equal amounts of proteins were fractionated in a polyacrylamide Bis-Tris 4–12% gradient gels (Invitrogen, NP0321PK2). After transfer to poly-vinylidene difluoride (PVDF) membranes (Millipore), the blots were blocked in Tris-buffered saline containing 5% non-fat milk and 0.1% Tween-20 and incubated with primary antibodies either for 1 h at room temperature or overnight at 4°C followed by incubation for 1 h with the appropriate horseradish peroxidase-conjugated secondary antibodies. The complexes were visualized by chemiluminescence (ECL; Pierce, 32,106).

## In vitro *affinity isolation assays*

BPLF1 production and purification in *E. coli* was previously described [64] and purified SQSTM1/p62 produced in *E. coli* was purchased from Abcam (ab95320). Proteins were mixed in approximately 1:1 molar ratio in binding buffer (50 mM Tris-HCl pH 7.6, 150 mM NaCl, 5 mM MgCl_2_, 1 mM EDTA, 1 mM DTT, 1% Igepal, 10% glycerol) supplemented with protease and phosphatase inhibitors from Roche (04693116001, 4906837001) and incubated for 30 min. in rotation. Equal aliquots of the mixture were incubated with antibodies specific for SQSTM1/p62, HIS-tag or rabbit or mouse isotype controls for 2 h in rotation followed by 1 h incubation with protein-G coupled Sepharose beads (GE Healthcare, 17–0885-01). After washing in binding buffer, the bound proteins were eluted by boiling in 2x reducing LDS sample buffer for 5 min.

### Filter trap assay

Cells were harvested in medium 24 or 48 h after transfection or treatment. Cell pellets were resuspended in PBS (Biowest, X0515-500) containing protease inhibitor cocktail. Samples were snap frozen and stored at −20°C. Lysates were sonicated with a QSonica Q125 sonicator with settings 20% amplitude, pulsating 1s on/1s off total time of 30 sec. After sonication protein amounts were measured with Bio-Rad Lowry kit according to manufacturer instructions. Samples with equal protein concentrations were prepared by diluting in PBS containing protease inhibitor cocktail and SDS was added to a final concentration of 1%. The lysates were suctioned through a cellulose acetate membrane (GE health care, 10,404,180) using a Bio-Rad Bio-Dot apparatus. Images were analyzed with the ImageJ software.

### Immunofluorescence and confocal microscopy

HeLa and U2OS cells were grown to semi-confluency in Dulbecco’s modified Eagle’s medium (Sigma, D6429) containing 10% fetal calf serum and 10 μg/ml ciprofloxacin on glass cover slips and transfected with the indicated plasmids using the JetPEI or Lipofectamine 2000 kit as recommended by the manufacturers. After treatment for the indicated time the cells were fixed in 4% paraformaldehyde (Merck, 100496) and permeabilized with cold methanol at −20°C for 2 min or 0.1% TX-100 in PBS for 5 min RT followed by blocking with 0.12% glycine (Fisher Scientific, G46-1) in PBS for 10 min. Blocking in 3% bovine serum albumin (BSA, Sigma, A7906) in PBS for 15 min at room temperature was performed before antibody labeling. The cells were labeled in 3% BSA-PBS using rabbit anti-LC3, mouse anti-SQSTM1/p62 and goat anti-FLAG or mouse anti-FLAG antibodies followed by the appropriate Alexa Fluor 488, 555, 594 or 647 conjugated secondary antibodies. The nuclei were stained with 2.5 μg/ml DAPI (Sigma, D9542) in PBS for 10 min and the coverslips were mounted cell side down on object glasses with Mowiol (Calbiochem, 475904) containing 50 mg/ml 1,4-diazabicyclo[2.2.2]octane (Dabco; Sigma, D-2522) as anti-fading agent. The samples were imaged using a confocal scanning laser microscope (Zeiss LSM880) and 1 μm optical sections were acquired.

### Image analysis

Images were analyzed with the Fiji software. The number of aggregates was counted with the analyze particles function. Colocalization of LC3 and SQSTM1/p62 labels was analyzed by creating a mask by thresholding the SQSTM1/p62 image and measuring fluorescence from LC3 image through the mask, acquiring a mean gray value for each structure. Empty-vector transfected sample was used as a control. Changes in the level of LC3 colocalization with SQSTM1/p62 structures were assessed by comparing the gray values in BPLF1- and BPLF1[C61A]-expressing cells with the mean gray value of all structures detected in empty-vector transfected cells.

### Statistical analysis

Student’s t test was used to evaluate the significance of differences between samples as detailed for each figure separately. P > 0.05 was considered statistically non-significant (ns), P < 0.05 = *, P < 0,01 = **; P < 0,001 = ***.

## Supplementary Material

Supplemental Material
