# Supplementary material for: The Epstein-Barr virus deubiquitinase BPLF1 targets SQSTM1/p62 to inhibit selective autophagy
Source: Autophagy. 2021 Jan 28;17(11):3461–74. doi: 10.1080/15548627.2021.1874660 (PMC8632276; doi:10.1080/15548627.2021.1874660)
Supplement: Supplemental Material [file KAUP_A_1874660_SM0777.zip › Supplementary information/KAUP-2020-0512-R4-Supplementary Information.docx]

**Supplementary Information**

**Figure S1.** Immunoprecipitated ubiquitinated SQSTM1/p62 is deubiquitinated by recombinant BPLF1. HeLa cells were transfected with HA-ubiquitin and BPLF1[C61A] to induce the ubiquitination of SQSTM1/p62. Endogenous SQSTM1/p62 was immunoprecipitated in denaturing conditions to disrupt non-covalent protein interactions and divided into three aliquots. One aliquot was resuspended in washing buffer and the remaining two were resuspended in washing buffer supplemented with 1:1000 or 1:10000 v:v of recombinant BPLF1 followed by incubation for 30 min at 37°C.

**Figure S2.** BPLF1 inhibits the recruitment of LC3 to SQSTM1/p62-positive structures in U2OS cells. (**A**) U2OS cells expressing catalytically active or inactive BPLF1 or empty vector control were stained for endogenous SQSTM1/p62 and LC3. In cells expressing catalytically active BPLF1 a significant decrease of LC3 recruitment to SQSTM1/p62-positive structures was observed. (**B**) Quantification of data represented in A. A minimum of 57 cells were scored in each sample.

**Figure S3.** The HTTQ103 aggregates are cleared by autophagy. The clearance of HTTQ103-CFP aggregates is inhibited by treatment with the autophagy inhibitor 3MA. (**A**) Representative western blots. (**B**) Quantification of three independent experiments. (**C**) HeLa cells were co-transfected with plasmids expressing HTTQ109-GFP, FLAG-BPLF1 and increasing amount of HA-SQSTM1/p62 and cell lysates collected after 48 h were analyzed in filter trap assay and immunoblotting. Representative immunoblots illustrating the capacity of HA-SQSTM1/p62 to rescue the accumulation of HTTQ109-GFP aggregates in a dose-dependent manner. (**D**) Quantification of data presented in C; means ± SD of two independent experiments.

**Table S1.** Functional annotation of the BPLF1-interacting proteins involved in autophagy and vesicular trafficking.

| ATG9A | autophagy related 9A; involved in autophagy and cytoplasm-to-vacuole targeting (Cvt) vesicle formation. Plays a key role in the organization of the phagophore assembly site (PAS), the nucleating site for formation of the sequestering vesicle. Cycles between a juxta-nuclear trans-Golgi network compartment and late endosomes. Nutrient starvation induces accumulation on autophagosomes. Starvation-dependent trafficking requires ULK1, ATG13 and SUPT20H (839 aa). |
| --- | --- |
| SQSTM1 | sequestosome 1; autophagy receptor that interacts directly with both the cargo to become degraded and an autophagy modifier of the MAP1LC3 family. Along with WDFY3, involved in the formation and autophagic degradation of cytoplasmic ubiquitin-containing inclusions (SQSTM1/p62 bodies, ALIS/aggresome-like induced structures). Along with SQSTM1, required to recruit ubiquitinated proteins to PML bodies in the nucleus. May regulate the activation of NFKB1 by TNF, NGF (nerve growth factor) and IL1 (440 aa). |
| BAG3 | BAG cochaperone 3; co-chaperone for HSPA/HSP70 and HSPA8/HSC70 chaperone proteins. Acts as a nucleotide-exchange factor (NEF) promoting the release of ADP from the HSPA/HSP70 and HSPA8/HSC70 proteins thereby triggering client/substrate protein release. Nucleotide release is mediated via its binding to the nucleotide-binding domain (NBD) of HSPA8/HSC70 where the substrate release is mediated via its binding to the substrate-binding domain (SBD) of HSPA8/HSC70. Has anti-apoptotic activity. Plays a role in the HSF1 nucleocytoplasmic transport; BCL2 associated athanogene family (575 aa). |
| BAG6 | BAG cochaperone 6; the corresponding gene was first characterized as part of a cluster of genes located within the human major histocompatibility complex class III region. This gene encodes a nuclear protein that is cleaved by CASP3 and is implicated in the control of apoptosis. In addition, the protein forms a complex with EP300 (E1A binding protein p300) and is required for the acetylation of TP53 in response to DNA damage. Multiple transcript variants encoding different isoforms have been found for this gene. |
| LAMP2 | Lysosomal associated membrane protein 2; plays an important role in chaperone-mediated autophagy, a process that mediates lysosomal degradation of proteins in response to various stresses and as part of the normal turnover of proteins with a long biological half-life. Functions by binding target proteins, such as GAPDH and MLLT11, and targeting them for lysosomal degradation. Plays a role in lysosomal protein degradation in response to starvation. Required for the fusion of autophagosomes with lysosomes during autophagy (411 aa). |
| AP1M1 | adaptor related protein complex 1 subunit mu 1; subunit of clathrin-associated adaptor protein complex 1 that plays a role in protein sorting in the trans-Golgi network (TGN) and endosomes. The AP complexes mediate the recruitment of clathrin to membranes and the recognition of sorting signals within the cytosolic tails of transmembrane cargo molecules (435 aa). |
| AP1S1 | adaptor related protein complex 1 subunit sigma 1 (158 aa). |
| AP2A1 | adaptor related protein complex 2 subunit alpha 1; component of the adaptor protein complex 2 (AP-2). Adaptor protein complexes function in protein transport via transport vesicles in different membrane trafficking pathways. Adaptor protein complexes are vesicle coat components and appear to be involved in cargo selection and vesicle formation. AP-2 is involved in clathrin-dependent endocytosis in which cargo proteins are incorporated into vesicles surrounded by clathrin (clathrin-coated vesicles, CCVs) which are destined for fusion with the early endosome. The clathrin lattice serves as a mechanical scaffold (977 aa). |
| AP2A2 | adaptor related protein complex 2 subunit alpha 2; component of the adaptor protein complex 2 (AP-2) (940 aa). |
| AP3B1 | adaptor related protein complex 3 subunit beta 1; subunit of non-clathrin- and clathrin-associated adaptor protein complex 3 (AP-3) that plays a role in protein sorting in the late-Golgi/trans-Golgi network (TGN) and/or endosomes. The AP complexes mediate both the recruitment of clathrin to membranes and the recognition of sorting signals within the cytosolic tails of transmembrane cargo molecules. AP-3 appears to be involved in the sorting of a subset of transmembrane proteins targeted to lysosomes and lysosome-related organelles. In concert with the BLOC-1 complex, AP-3 is required to target cargos (1094 aa). |
| USO1 | USO1 vesicle transport factor; required for inter-cisternal transport in the Golgi stack; it is required for transcytotic fusion and/or subsequent binding of the vesicles to the target membrane. May well act as a vesicular anchor by interacting with the target membrane and holding the vesicular and target membranes in proximity; armadillo-like helical domain containing (962 aa). |
| RAB7A | RAB7A, member RAS oncogene family; key regulator in endo-lysosomal trafficking. Governs early-to-late endosomal maturation, microtubule minus-end- as well as plus-end-directed endosomal migration and positioning, and endosome-lysosome transport through different protein-protein interaction cascades. Plays a central role, not only in endosomal traffic, but also in many other cellular and physiological events, such as growth-factor-mediated cell signaling, nutrient-transporter-mediated nutrient uptake, NTF (neurotrophin) transport in the axons of neurons and lipid metabolism (207 aa). |
| ARF4 | ADP ribosylation factor 4; GTP-binding protein that functions as an allosteric activator of the cholera toxin catalytic subunit, an ADP-ribosyl transferase. Involved in protein trafficking; may modulate vesicle budding and uncoating within the Golgi apparatus; ARF GTPase family (180 aa). |
| GBF1 | golgi brefeldin A resistant guanine nucleotide exchange factor 1; guanine-nucleotide exchange factor (GEF) for members of the ARF family of small GTPases involved in trafficking in the early secretory pathway; its GEF activity initiates the coating of nascent vesicles via the localized generation of activated ARFs through replacement of GDP with GTP. Recruitment to cis-Golgi membranes requires membrane association of ARF-GDP and can be regulated by ARF1, ARF3, ARF4 and ARF5. Involved in the recruitment of the COPI coat complex to the endoplasmic reticulum exit sites (ERES) (1859 aa). |
| PDCD6 | programmed cell death 6; calcium sensor that plays a key role in processes such as endoplasmic reticulum (ER)-Golgi vesicular transport, endosomal biogenesis or membrane repair. Acts as an adapter that bridges unrelated proteins or stabilizes weak protein-protein complexes. Involved in ER-Golgi transport by promoting the association between PDCD6IP and TSG101 (191 aa). |
| PDCD6IP | programmed cell death 6 interacting protein; multifunctional protein involved in endocytosis, multivesicular body biogenesis, membrane repair, cytokinesis, apoptosis and maintenance of tight junction integrity. Class E VPS protein involved in concentration and sorting of cargo proteins of the multivesicular body (MVB) for incorporation into intralumenal vesicles (ILVs) that are generated by invagination and scission from the limiting membrane of the endosome. Binds to the phospholipid lysobisphosphatidic acid (LBPA) which is abundant in MVB internal membranes (873 aa). |
| ANXA11 | annexin A11; binds specifically to calcyclin in a calcium-dependent manner (by similarity). Required for midbody formation and completion of the terminal phase of cytokinesis; belongs to the annexin family (505 aa). |
| HGS | hepatocyte growth factor-regulated tyrosine kinase substrate; involved in intracellular signal transduction mediated by cytokines and growth factors. When associated with STAM, it suppresses DNA signaling upon stimulation by IL2 and CSF2/GM-CSF. Could be a direct effector of PtdIns 3-kinase in vesicular pathway via early endosomes and may regulate trafficking to early and late endosomes by recruiting clathrin. May concentrate ubiquitinated receptors within clathrin-coated regions. Involved in downregulation of receptor tyrosine kinase via multivesicular bodies (MVBs) when complexed with STAM (777 aa). |
| EGFR | epidermal growth factor receptor tyrosine kinase, binding ligands of the EGF family and activating several signaling cascades to convert extracellular cues into appropriate cellular responses. Known ligands include EGF, TGFA/TGF-alpha, AREG (amphiregulin), EPGN/epigen, BTC (betacellulin), EREG (epiregulin) and HBEGF (heparin binding EGF like growth factor). Ligand binding triggers receptor homo- and/or heterodimerization and autophosphorylation on key cytoplasmic residues. The phosphorylated receptor, recruits adaptor proteins such as GRB2, which in turn activates complex downstream signaling cascades. (1210 aa). |
